# Editorial: Disease modifying therapies in multiple sclerosis

**DOI:** 10.3389/fneur.2022.927321

**Published:** 2022-08-22

**Authors:** Simona Bonavita, Mahsa Ghajarzadeh, Luigi Lavorgna

**Affiliations:** ^1^Dipartimento di Scienze Mediche e Chirurgiche Avanzate, Università della Campania Luigi Vanvitelli, Napels, Italy; ^2^Universal Council of Epidemiology (UCE), Universal Scientific Education and Research Network (USERN), Tehran University of Medical Sciences, Tehran, Iran

**Keywords:** multiple sclerosis, disease modifying therapy, predictors, pregnancy, pediatric onset multiple sclerosis

Multiple sclerosis (MS) is a chronic autoimmune neuroinflammatory disorder of the central nervous system (CNS); it has an increasing prevalence worldwide and preferentially affects women of childbearing age.

Since the introduction of the first disease-modifying treatment (DMT), in the early 90's, numerous compounds have been developed, posing new challenges to the choice of the most appropriate therapeutic strategy for the individual patient with MS.

For this reason, there has been increasing efforts in developing decisional algorithms to stratify patients based on their clinical and radiological characteristics; more recently, with the Covid-19 pandemic, DMT choice has become even more difficult as clinicians attempt to balance the benefit with the infection risk potentially amplified by certain drugs.

In this Research Topic, we focused on potential drugs for MS, available DMTs, their efficacy and safety profiles, during the Covid-19 pandemic, in patients with different levels of disability, and particular conditions such as pediatric age and pregnancy.

Radandish et al. reviewed the pathogenetic role of microglia in MS and the potential effect of drugs targeting it. In the early stage of experimental autoimmune encephalomyelitis (EAE) and MS, the pro-inflammatory microglia (M1) has different roles in the promotion of inflammation through cytokine/chemokine release, and ROS and NO production lead to demyelination, thus the suppression of M1 can be useful in MS control. Several drugs (i.e., galectin-1, TQ, and Que) may act against the activated microglia, inhibiting the release of pro-inflammatory cytokines; others (i.e., FTY-720) suppress microglial activation and promote the switch from M1 to M2 (anti-inflammatory) phenotype. Conversely, M2 has anti-inflammatory functions and promotes remyelination *via* cytokines release; therefore, other potential drugs promoting M2 activity (IL-4, activin-A, IVM, rHIgM22, and rIFN-b, M-CSF, and progesterone) may potentially benefit EAE or MS.

Based on the results by Yang and Shi on experimental models of MS, other therapeutic targets, such as dendritic cells, could potentially prompt further studies on new molecules; indeed, these authors demonstrated a beneficial effect of silybin on EAE by inhibition of dendritic cell activation and Th17 cell differentiation. Silybin, blocking the migration of inflammatory cells into the CNS and remarkably inhibiting the demyelinating process, can relieve the disease development.

Ceylan et al. investigated *in vitro* the effects of iron on microglia and used the antipsychotic clozapine *in vitro* and chronic EAE to target features of progressive MS and identify protective medications. These authors found that iron impaired microglial function *in vitro*, while clozapine was able to regulate this effect by reducing the release of IL-6 and by normalizing neuronal phagocytosis. In chronic EAE, clozapine dose-dependently attenuated clinical signs and still had an effect if applied in the therapeutic setting. Histologically, demyelination was reduced by clozapine, and positive effects on inflammation strongly correlated with reduced iron deposition. These data deserve attention because they suggest that clozapine might be considered a possible add-on therapeutic for further development in progressive MS.

Moving on from EAE to MS, the pathogenetic role of intestinal permeability (IP) has been investigated by Buscarinu et al., also in relation to treatment with dimethyl fumarate (DMF). The authors focused on the gut triggers that may lower the threshold for disease development in susceptible individuals and investigated IP changes, the circulating CD161+CD8+ T-cell subset, and clinical/neuroradiological data in a cohort of relapsing-remitting (RR) MS patients before and after 9 months of DMF therapy. At baseline, 64% patients showed altered IP, while 56% had an active MRI. During DMF therapy they found a reduction in the percentage of CD161+CCR6+CD8+ T cells that significantly correlated with IP changes and a drop in MRI activity.

Tobin et al. reviewed the data supporting the role of gut microbiota and short-chain fatty acid (SCFA) metabolites, in particular propionate, in the pathophysiology of MS. Dysbiosis is responsible for a reduction in SCFA producing bacteria and in MS patients a reduction in stool and plasma levels of propionate has been shown. In particular, the action of propionate on T-cell activity results in decreased Th1 and Th17 pro-inflammatory profile and increased regulatory T cell and an overall anti-inflammatory profile, supporting the clinical benefit induced by supplementation of propionate in MS patients.

Treatment strategies are still a matter of debate; however, there is increasing evidence that the first choice in the clinical history of MS patients might deeply impact their future disability. This is the direction Simonsen et al. take, by using a real-world population-based registry to examine the impact of initial treatment in achieving no evidence of disease activity (NEDA) in patients treated with moderate or high efficacy DMTs. Their results showed that NEDA at year 1 and 2 is significantly more likely in patients on high-efficacy DMTs than on moderate efficacy therapies (68 vs. 36% year 1, 52.4 vs. 19.4% year 2), and the first choice of treatment is the most important.

Real-world studies on the efficacy and safety of DMTs are of great value to help MS neurologists in their clinical practice.

Boziki et al. reported the real-world experience of a Greek MS center about the efficacy and safety of natalizumab (NTZ) and fingolimod (FTY) in patients with long-term follow-up. In the matched analysis, NTZ was superior to FTY either for time to first relapse or for time to MRI activity under treatment and treatment discontinuation due to MRI activity. The safety profile of the two drugs confirmed the results from registration trials.

Ziemssen, Albrecht et al. investigated the effectiveness of FTY in young adults ( ≤ 20 and >20 to ≤ 30 years) compared to older patients (>30 years) enrolled in the PANGAEA study. Although young adults had higher annual relapse rates (ARR) at study entry, the proportion of patients with no clinical disease activity in year 4 was significantly higher in young patients compared to older ones. Moreover, in the long-term follow-up, cognitive performances improved more in young adults than in older ones. These data suggest that young age is the best age frame for FTY treatment.

Ziemssen, Hoffmann et al. also reported the results of the interim analysis of the TREAT-MS study collecting data on the long-term effectiveness and safety of alemtuzumab in a large real-life cohort of MS patients. In non-naive patients, treatment sequences were documented, showing that patients with longer disease duration and higher EDSS had a higher number of previous DMTs. Compared to those enrolled in the registration trials, patients in the TREAT-MS study had a longer disease duration and a variety of previous DMTs. Effectiveness and safety data from this study, as well as patients' characteristics, might be useful to support future treatment decisions.

In clinical practice, safety concerns very often prompt the off-label use of DMTs, therefore real-life studies become relevant to understand whether drug effectiveness is preserved. In this regard, Riancho et al. reported the results of a 7-Year Retrospective Observational Study aimed to analyze the efficacy and safety of treatment with NTZ in MS patients initially treated with standard interval dosing (SID) who were then switched to extended interval dosing (EID) every 8 weeks. ARR, radiological activity, and disability progression did not significantly vary between the SID and EID groups. Furthermore, the proportion of patients maintaining the NEDA-3 status was slightly higher among naïve patients than among switchers, suggesting that earlier use of NTZ may benefit active patients.

Proschmann et al. characterized the pharmacokinetics and -dynamics and serum neurofilament light chain (sNfL) in correlation to clinical data in patients with RRMS and secondary progressive MS (SPMS) stopping NTZ. The authors measured free NTZ concentration, cell-bound NTZ, α4-integrin expression, and α4-integrin-receptor saturation as well as immune cell frequencies for up to 4 months after NTZ withdrawal. Additionally, sNfL levels were observed for up to 12 months in RRMS and up to 4 months in SPMS patients. After stopping NTZ, disease activity returned in 38% of the RRMS and 33% of the SPMS patients within 12 and 7 months, respectively. The concentration of free and cell-bound NTZ, as well as α4-integrin-receptor saturation, decreased in the RRMS and SPMS patients whereas α4-integrin expression increased over time. In all RRMS during the follow-up period, sNfL levels peaked up to 16-fold and were linked to the return of disease activity in more than 50% of patients. This relation was observed also at the individual level; therefore, the authors suggest that they can also serve in clinical practice as an early marker to predict the recurrence of clinical or radiological disease activity.

Clinical response to DMTs varies among people with MS and within the same patient in different moments of their MS history. The identification of biomarkers to early identify responders to the different DMTs is a field of active research; Devi-Marulkar et al. investigated the cellular and molecular blood signatures associated with the efficacy of IFNb treatment by phenotyping regulatory CD4+ T cells and naïve/memory T cell subsets, by measuring the circulating IFNa/b proteins, and by analyzing ~600 immune-related genes, including 159 interferon-stimulated genes. They also investigated the potential impact of HLA class II gene variation in treatment responsiveness by genotyping HLA-DRB1, -DRB3,4,5, -DQA1, and -DQB1. Non-responders had reduced circulating naïve regulatory T cells, enhanced effector memory CD4+ TEMRA cells, and altered expression of at least six genes with immunoregulatory function. Moreover, non-responders were enriched for HLA-DQB1 genotypes encoding DQ8 and DQ2 serotypes. All these data suggest that IFNb non-responders may suffer from pathogenic CD4+ T cells, likely restricted by DQ8 and DQ2, that may exert autoreactive and bystander inflammatory activities.

The study by Lorefice et al. aimed to characterize MS patients exposed to DMF to evaluate the predictors of therapeutic response. In this observational monocentric study, the authors examined the prescription flow of DMF in MS patients from 2015 to 2019 and analyzed clinical and MRI data and NEDA-3 status at 24 months of DMF treatment. Predictors of DMF response were lower ARR in 2 years pre-treatment and being naive patients; these parameters were associated with the NEDA-3 status at 24 months. A good efficacy profile of DMF was demonstrated in both naive patients and horizontal switchers although it did not eliminate the risk of MS reactivation in patients previously treated with NTZ.

Although siponimod was recently approved for secondary progressive MS, the treatment for patients with the progressive disease has been a challenge for a long time. Indeed, despite the development of highly efficient immunotherapies for MS, no treatment can completely suppress the compartmentalized and meningeal inflammation in the CNS that drives tissue injury and disability progression, and effectively promote regeneration–remyelination. Stem cells are strong immunomodulators that may potentially downregulate the localized and compartmentalized inflammation and may induce neuroprotection and enhance endogenous remyelination (as indicated by animal studies). In this Research Topic, we report the results by Petrou et al. who evaluated the safety and the long-term clinical and immunological effects of multiple intrathecal (IT) and intravenous (IV) injections (up to 8) of autologous mesenchymal stem cells (MSCs) in 24 patients with active-progressive MS at intervals of 6–12 months, followed up for 4 years. In general, there were no serious side effects and most of the patients were stable or improved at the last follow-up visit. Immunological follow-up showed a transient upregulation of CD4+CD25+FoxP3+ cells and downregulation of the proliferative ability of lymphocytes, sustaining the hypothesis that MSCs effects are mediated through peripheral immunomodulation. Since the authors recently demonstrated that the IT injection of MSC was superior to the IV at several parameters, they advocate that the neuroprotective and neurotrophic mechanisms play the most crucial role.

A further challenge in the treatment of MS is represented by pediatric patients (POMS) and pregnant women. Margoni et al. reviewed the state of the art in POMS therapy; observational and clinical studies on first-line and second-line immunomodulatory therapies in POMS have been reported. Since POMS is a severe form of MS, characterized by a high clinical and radiological activity and younger age at reaching cognitive and physical disability milestones, second-line treatment is preferred as demonstrated by the fact that the off-label use of newer DMTs is increasing in POMS and retrospective studies, case series, and phase II trials indicate that this approach appears to be highly effective and safe in children.

Lastly, Simone et al. collected the current evidence on the influence that pregnancy has on MS and the resulting impact of DMTs. Additionally, they discussed safety profiles for each drug and correlated them to both risks for the exposed fetus and risk for the mother interrupting treatments when seeking pregnancy. Based on current evidence, MS does not impact fertility or the women's ability to carry the fetus to term. The disease does not increase the risk of spontaneous abortion, malformations, and cesarean delivery. Pregnancy does not impact the long-term accumulation of disability, rather it appears to be protective against disease activity, particularly during the third trimester, but an increased risk of relapse is reported in the first 3 months postpartum. Exclusive breastfeeding may have a possible favorable effect. Since evidence suggest that some drugs could be safely used throughout the whole pregnancy course or, in specific cases, till the third trimester, neurologists should tailor the best therapy for any pregnant woman, without exposing the fetus to any possible risk and the mother to disease reactivation both in pregnancy and in the postpartum period.

## Author contributions

SB wrote the editorial. MG and LL approved the draft. All authors contributed to the article and approved the submitted version.

## Conflict of interest

The authors declare that the research was conducted in the absence of any commercial or financial relationships that could be construed as a potential conflict of interest.

## Publisher's note

All claims expressed in this article are solely those of the authors and do not necessarily represent those of their affiliated organizations, or those of the publisher, the editors and the reviewers. Any product that may be evaluated in this article, or claim that may be made by its manufacturer, is not guaranteed or endorsed by the publisher.

